# Supplementary material for: Static network structure cannot stabilize cooperation among large language model agents
Source: PLoS One. 2025 May 22;20(5):e0320094. doi: 10.1371/journal.pone.0320094 (PMC12097601; doi:10.1371/journal.pone.0320094)
Supplement: SI Appendix — This supporting file shows the dialogue generated from an experiment. [file pone.0320094.s001.pdf]

Static network structure cannot stabilize cooperation among Large Language Model agents

Jin Han[1], Balaraju Battu[2], Ivan Romić[3], Talal Rahwan[2], and Petter Holme[1,3,*]

[1]Department of Computer Science, Aalto University, 00076 Aalto, Finland
[2]New York University Abu Dhabi, United Arab Emirates
[3]Center for Computational Social Science, Kobe University, Kobe, Hyogo 657-8501, Japan
[*]petter.holme@aalto.fi

ABSTRACT

This Supplementary Information shows an example of the kind of dialogue with an LLM that provides the experimental data for our study.

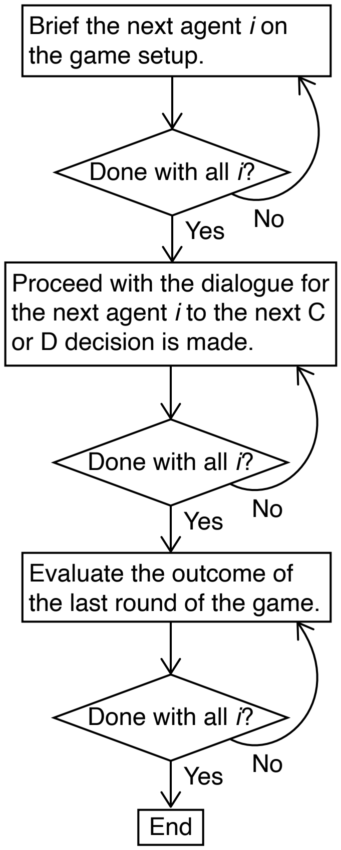

Figure 1. An illustration for one run replicating one experiment in the data collection phase.

The structure of data collection

The data collection follows the same structure as a lab experiment, except that the interaction with the LLM agents is not actually in parallel. In Fig. 1, we show a schematic illustration of the protocol.

An example of a dialogue with the LLM

Here follows the beginning of a dialogue representing one run of an experiment. The longer instructional texts are adapted from Ref. 1.

— Pretend you are a human playing a prisoner's dilemma game in a lab environment. I will now explain the rules to you, are you ready?

— Yes, I am ready to learn the rules of the prisoner's dilemma game. Please go ahead.

— The game will be played over a series of rounds. In every round, you make a choice about whether to pay to give points to the other players you are connected to. The game will last for about 15 rounds. You will be connected to the same people every round. We now describe the game in more detail. Are you ready?

— Yes, I am ready to learn more details about the prisoner's dilemma game. Please proceed.

— In every round, you choose whether to pay to give points to the people you are connected to. If you choose to cooperate (represented by "C"), you pay 10 points for each player you are connected to, and each of them gains 20 points. If you choose to defect (represented by "D"), you do not pay any points and do not change the points of the players you are connected to. Each player you are connected to has the same choice. For each of them that chooses "C," you gain 20 points. Once everyone makes a decision, I will tell you the result of the round. You will be shown the choices of each player you are connected to and how many points in total you gained or lost. You will also be shown how many points each player you are connected to gained or lost in total. These numbers are affected by your choice, their choice, and also the choices of any other players connected by them who may or may not be connected to you. Remember, for every 300 points you have at the end of the game, we will pay you 1 dollar. You should aim to get as many points as you can. Do you understand?

— Yes, I understand the rules of the prisoner's dilemma game.

— You have now completed the tutorial. Are you ready to play the game?

— Yes, I am ready to start playing the prisoner's dilemma game. Let's begin!

— Thank you for completing the tutorial. You will now be playing with other participants. Please only give your choice with "D" or "C." Are you ready?

— I understand the rules of the game, and I am ready to participate. Please proceed.

— Please make a choice for the first round. Please only reply with "D" or "C."

— C.

— Last round, you chose C, paid 20 points, and gained 40 points, for a total of 20 points. Neighbor 1 chose C, paid 20 points, and gained 20 points from you and 20 points from other players, for a total of 20 points. Neighbor 2 chose C, paid 20 points, and gained 20 points from you and 0 points from other players, for a total of 0 points. Please choose D or C for the current round.

— C.

And so on.

References

1. Rand, D. G., Nowak, M. A., Fowler, J. H. & Christakis, N. A. Static network structure can stabilize human cooperation. *Proc. Natl. Acad. Sci. USA* **111**, 17093–17098 (2014).
